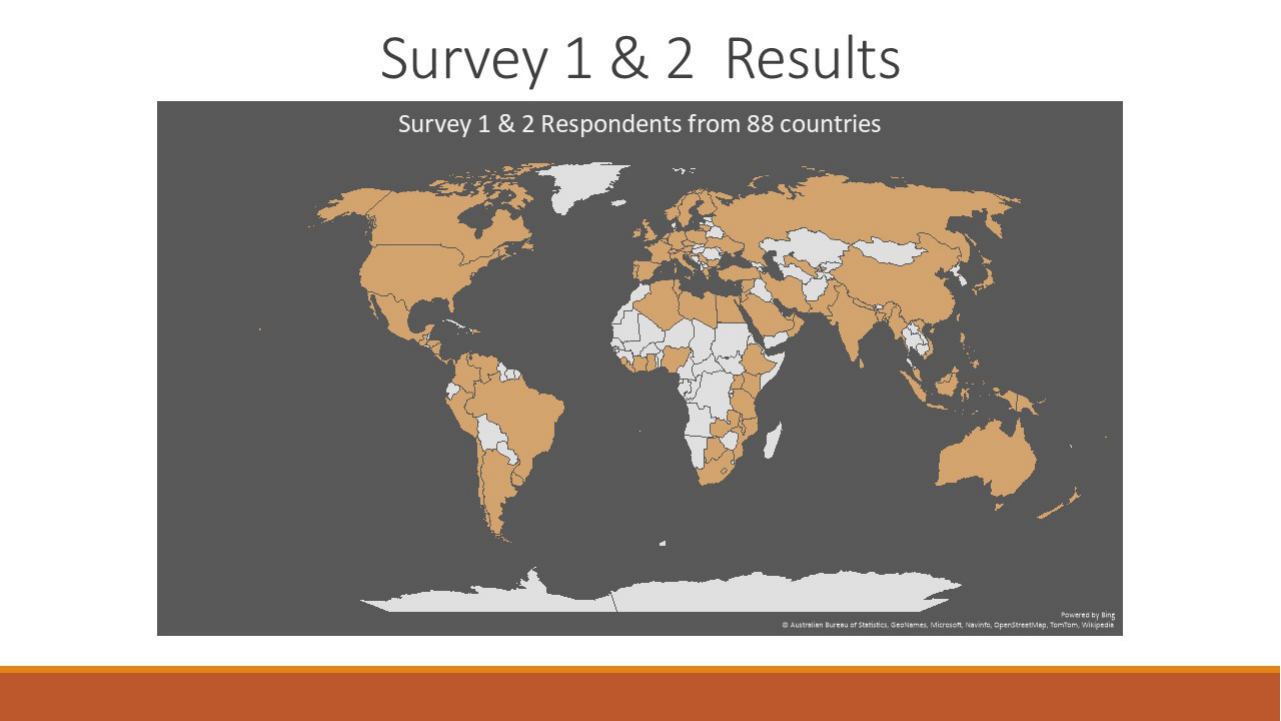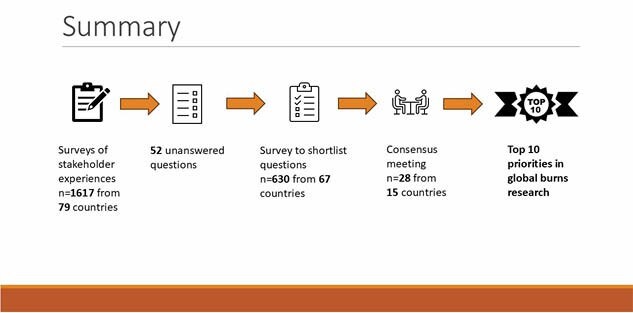# 946 Involving Burn Survivors and Caregivers in Research Prioritization: Recommendations for Community-Based Participatory Research in Burns

**DOI:** 10.1093/jbcr/iraf019.477

**Published:** 2025-04-01

**Authors:** Hollie Richards, Jane Blazeby, Lise Deguire

**Affiliations:** National Institute of Health and Care Research Bristol Biomedical Research Centre; National Institute of Health and Care Research Bristol Biomedical Research Centre; Private Practice

## Abstract

**Introduction:**

Despite many advances, burns research doesn’t often address the issues that are most vital to stakeholders (e.g. survivors). Community-based participatory research (CBPR) ensures research is conducted with/by patients/public. The James Lind Alliance (JLA) Priorities in Global Burns Research Prioritisation Setting Partnership aimed to represent stakeholder’s needs by developing the top ten research priorities for global burns. We hypothesized that inclusion of burn survivors at each phase would substantially enhance the relevancy of findings for survivors and caregivers.

**Methods:**

The JLA uses a standardized methodology for identifying research priorities. Following the formation of a Steering Group (SG), we conducted multi-lingual online surveys to collate the lived experiences of burn survivors, caregivers, and HCPs. We undertook qualitative analysis to develop a long list of priorities which then prioritized in a second survey. A final consensus meeting determined the top ten. CBPR members of the SG provided oversight of the project, development of the surveys and analysis. Surveys were translated into 10 languages. CBPR work included collaboration with international support groups and proofreading of translations to ensure cultural competency. Survivors and caregivers were recruited to attend the final consensus workshop to ensure CBPR representation in the selection of the top ten.

**Results:**

The SG comprised of 30 members from 12 countries, including CBPR representatives who advised on terminology. Representatives of survivors’ support groups and international collaborators advised on the most appropriate means of survey distribution in their settings. CBPR SG members contributed to qualitative analysis. In total, survey responses were received from 88 countries. The final consensus workshop was attended by 28 participants from 15 countries, 14 of whom were CBPR representatives. This ensured that the top ten priorities were important to burn survivors.

**Conclusions:**

We have demonstrated the value of CBPR in research prioritization. Our recommendations for future work include CBPR involvement from the earliest stage of studies and at each phase, including developing methodology and qualitative analysis. Additionally, active engagement with existing survivor support groups is a way to include survivors and stakeholders in research.

**Applicability of Research to Practice:**

This work demonstrates the importance of involvement of stakeholders in research and burns care. By co-producing the Top 10 priorities in global burns research with survivors, caregivers, and healthcare professionals, while also providing a central voice for survivors, we have shown the value of CBPR. In terms of applicability to practice, our approach to CBPR can be incorporated into policy and treatment decisions, for example in service evaluations or patient pathway planning, to ensure the needs of survivors are central to all aspects of care.

**Funding for the Study:**

This work was funded by a National Institute for Health Research (UK) Advanced Fellowship